# Chronic Inflammatory Orbitopathy Hiding Orbital Lymphoma

**DOI:** 10.7759/cureus.23040

**Published:** 2022-03-10

**Authors:** Samah TAHRI, Habiba Alaoui, Houda Bachir, Siham Hamaz, Khalid Serraj

**Affiliations:** 1 Internal Medicine, University Mohammed First - Faculté of Medicine and Pharmacy, Oujda, MAR; 2 Immunohematology Cellular Therapy, Medical School, Oujda, MAR; 3 Infectious Diseases, Medical School, Oujda, MAR; 4 Internal Medicine, Medical School, Oujda, MAR

**Keywords:** chemotherapy, immunotherapy, radiotherapy, inflammatory orbitopathy, marginal zone lymphoma, primary orbital lymphoma

## Abstract

The incidence of lymphoma is constantly increasing worldwide. The reasons for this increase are unclear and likely multiple. B cell lymphomas represent the majority of non-Hodgkin lymphomas. Primary orbital localization remains a rare entity to think about in order to avoid missing a therapeutic emergency. In this article, we report the case of a 52-year-old man who has been treated for five years for an inflammatory orbitopathy with steroids, but the worsening of the clinical condition and the installation of exophthalmia led to push investigations and the outcome was a primary orbital lymphoma marginal zone type. The patient was treated by systemic chemotherapy with immunotherapy (RCHOP protocol) with a very good evolution and complete disappearance of the lesion after chemotherapy.

## Introduction

Lymphoma is a heterogeneous group of several subtypes of lymphoproliferative malignancies that are usually classified into two categories: Hodgkin lymphoma, and all other lymphomas into non-Hodgkin lymphoma (NHL). Primary ophthalmic malignancies are usually Non-Hodgkin’s lymphoma, with a rate of 1-2% of all NHL [1]. Depending on the site of involvement, ocular lymphoma can be either intraocular or orbital adnexal.

Ocular adnexal NHL can arise from the conjunctiva, lachrymal gland, orbit, or eyelid. Extra-nodal marginal zone B-cell lymphoma of mucosa-associated lymphoid tissue (MALT) type is the most common histologic type of primary ocular NHL [2]. The rate of occurrence of ocular adnexal NHL is equal in both genders with a predominance among Asians/Pacific islanders, in contrast to non-ophthalmic extra-nodal and nodal NHL which show predominance in males and Caucasians [3]. There is much debate in the literature about the role of pathogens in the predisposition to ocular adnexal NHL involving two species of Chlamydia (C. trachomatis and C. psittaci) [3].

## Case presentation

We report the case of a 52-year-old man with a long history of tobacco use who was treated over the course of five years for an inflammatory orbitopathy with corticosteroids without improvement. It's evolution was marked by the appearance of exophthalmia with decreased visual acuity.

**Figure 1:**
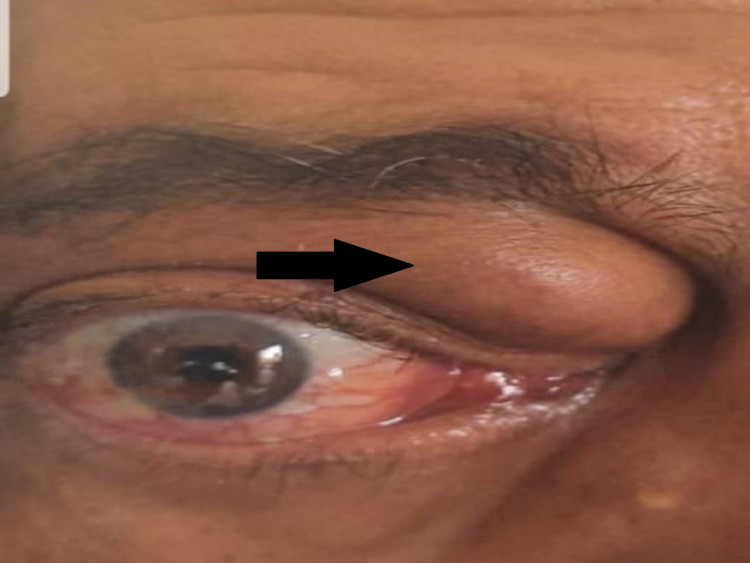
Tumor at the internal angle of the right eye (black arrow).

Physical examination found a painless eyelid swelling on the right eye’s internal angle with an important exophthalmia and excessive lachrymal non-purulent secretion. No inflammatory signs were found next to the tumor (Figure 1). Decreased visual acuity was reported by the patient. No tumor syndrome was found, including adenomegaly, hepatomegaly and splenomegaly.

**Figure 2:**
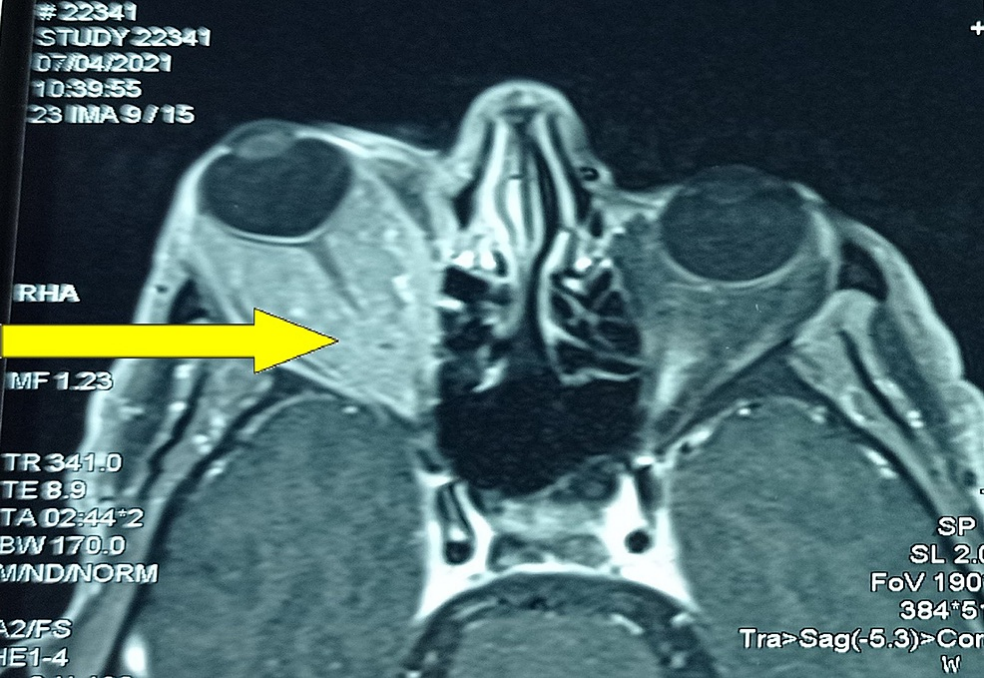
Orbital adnexal tumor (yellow arrow) that infiltrated orbital muscles and sheathed the optic nerve.

**Figure 3:**
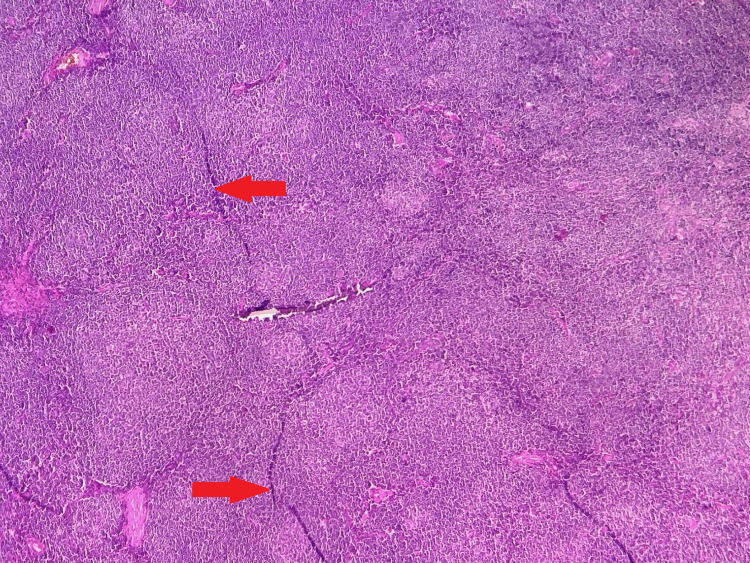
Microphotography showing a lymph node parenchyma whose architecture is erased and replaced by a nodular tumor proliferation surrounding preserved germinal centers (Red arrows) (HE, x40) HE: Hematoxylin and Eosin staining

**Figure 4:**
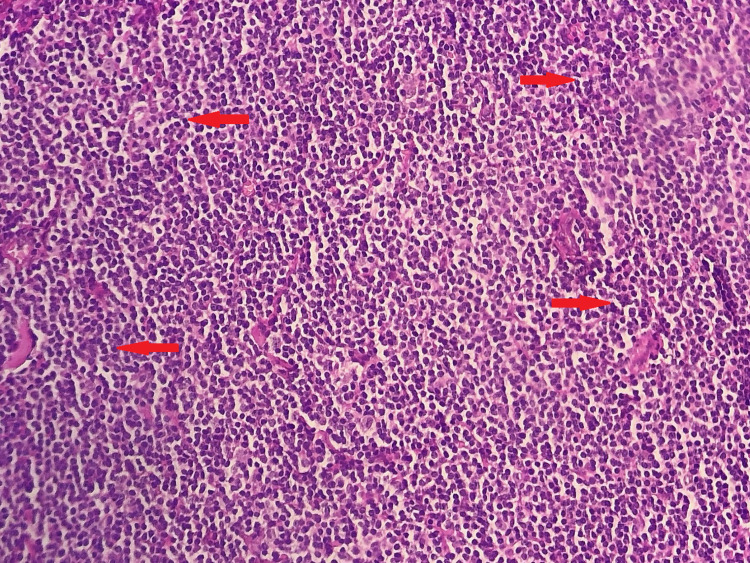
Tumor cells are small, with irregular contours, hyperchromatic nuclei, and scant clear cytoplasm (Red arrows showing examples) (HE, x200) HE: hematoxylin and eosin staining

**Figure 5:**
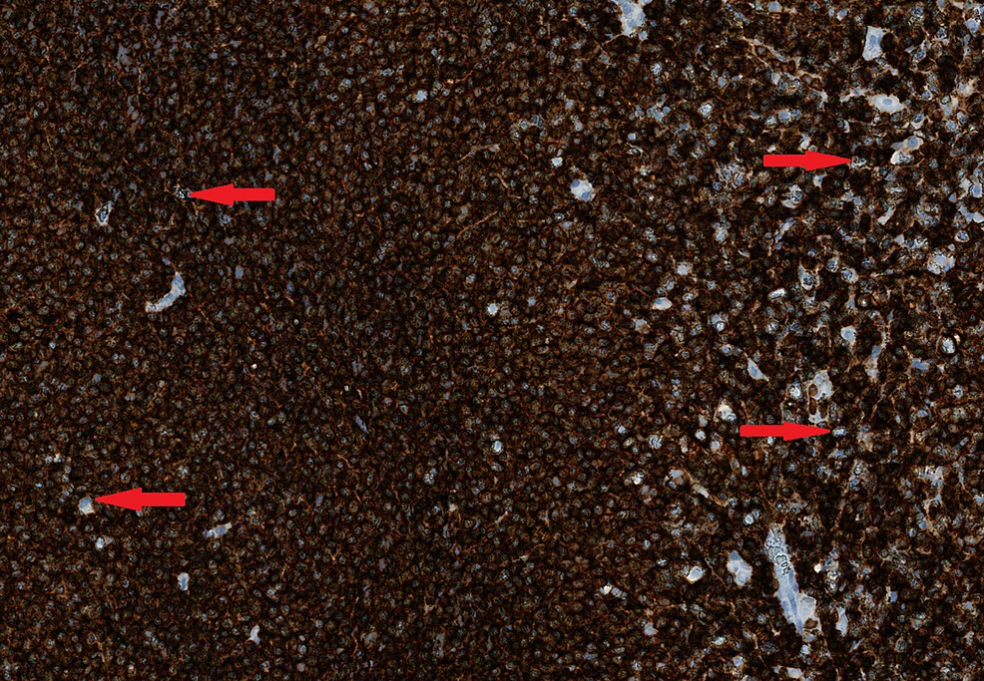
Tumor cells strongly and diffusely express CD20 (Red arrows showing examples). CD: cluster differentiation

Ophthalmological examination revealed a papillary edema of the right eye. Given the progressive nature of the disease, the patient benefited from an orbital magnetic resonance imaging (MRI) that revealed a right exophthalmia secondary to an extra-ocular intra-orbital lesion process that had infiltrated orbital muscles and encompassed the optic nerve (Figure 2). A biopsy of the orbital process was performed, and the histological examination revealed lymphomatous tumor proliferation in large nodules (Figure 3). The tumor cells were small with hyperchromatic nuclei and irregular contours and they were sometimes nucleolated. The cytoplasm was clear and not abundant. Rare figures of mitosis were noted (Figure 4). An immunohistochemical study was carried out, which showed that the tumor cells expressed CD20 (cluster differentiation) (Figure 5) and BCL2 (B-cell Lymphoma) and did not express BCL6, CD10, or cyclin D1. The proliferation index assessed by Ki67 was estimated at 15-20%, concluding to a marginal zone lymphoma.

Thoraco-abdominal-pelvic computed tomography (CT) scan, bone marrow biopsy, cerebral MRI, and lumbar puncture had eliminated a secondary localization of lymphoma. Thus, the disease was classified as a stage IEA according to Ann Arbor classification and T3N0M0 according to TNM (T: primary tumor, N: regional lymph nodes, M: distant metastasis) classification. Given the long-term evolution, the possibility of metastasis masked by corticotherapy and difficulty to access to PET scan (positron emission tomography) in our context, the patient was treated with systemic chemotherapy following the R-CHOP protocol every 21 days for six cycles (Table 1).

**Table 1:** R-CHOP regimen i.v: intravenous                p.o : per os

Drug	Dose	Mode	Day
Rituximab	375mg/m²	i.v	1
Cyclophosphamide	750mg/m²	i.v	1
Doxorubicin	50mg/m²	i.v	1
Vincristine	1.4mg/m² (≤2mg)	i.v	1
Prednisone	100mg	p.o	1-5

**Figure 6:**
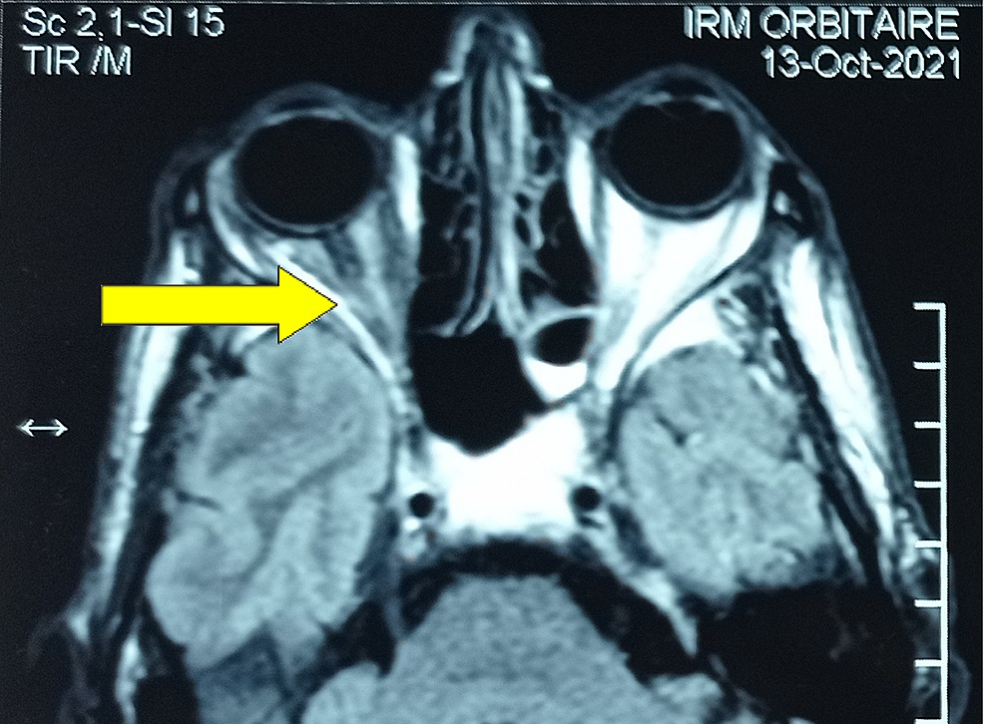
MRI imaging after six chemotherapy cycles that objectified more than 80% of response rate (yellow arrow).

A clinical and radiological response estimated superior than 80% in orbital control imaging after six cycles (Figure 6).

## Discussion

Typically, extra-nodal marginal zone lymphoma has an indolent clinical course and remains localized to the site of origin for a long time before dissemination [4]. The diagnosis is often delayed from one month to ten years due to multiple differential diagnoses [5].

The search for the etiologic agent of orbital inflammation should be accompanied by tissue biopsy obtained via a minimally invasive approach. The anatomopathological study should be interpreted in the context of clinical, radiological, biological, and serological findings, and the practice of a diagnostic trial based on corticosteroids administration should be abandoned [6].

An estimated 30% to 50% of patients with orbital lymphoma of all types present or will present a systemic disease, hence the interest in extension assessment with a computed tomography (CT) scan, bone marrow biopsy, and cerebrospinal fluid in high grade of malignancy [7]. It may take years for systemic involvement to be detected, which is why the long-term systemic follow-up is obligatory.

The modified classification of Ann Arbor is typically used to classify orbital lymphoma. However, it has multiple weaknesses as it does not take into consideration the following criteria: laterality, the contiguity or lack thereof in the orbital annexes sites and the involvement of extra nodal sites such as the parotid or sub mandibular galls. Therefore, the TNM (tumor, nodes, metastasis) classification was developed by the American Joint Committee on Cancer (AJCC) in 2009 to overcome these weaknesses and provide better precision of extension assessment [8].

Optimal treatment of the early stage orbital adnexal lymphoma is unclear because of the rare cases of early-stage case reports, incomplete staging, or pseudo tumors. A few studies, mostly retrospective, demonstrate that for low-grade lymphoma, radiotherapy alone may provide excellent results with a five-year progression-free survival of 100%. For patients with aggressive lymphoma, chemotherapy with or without radiotherapy has shown better results compared to radiotherapy alone. The combination of chemotherapy and radiotherapy has yielded better results than chemotherapy alone in terms of progression-free survival. Although studies on chemotherapy benefits in early-stage orbital lymphoma are rare, the R-CHOP regimen has proven efficiency in the treatment of extra-nodal marginal zone lymphoma among other chemotherapy associations with anti-CD20 antibody molecules [7].

## Conclusions

Orbital adnexal marginal zone lymphoma is a rare affliction. Biopsy with immunohistochemical study should be practiced in the etiological assessment of inflammatory orbitopathy. While the Ann Arbor classification has multiple weaknesses, the TNM classification is better adapted to classify orbital lymphoma. There is no treatment consensus concerning early-stage orbital lymphoma.

## References

[1] Fung CY, Nancy JT, Mark JL, Goldberg SI, Linggood RM, Harris NL, Ferry JA (2003). Ocular adnexal lymphoma: clinical behavior of distinct World Health Organization classification subtypes. Int J Radiat Oncol Biol Phys.

[2] Auw-Haedrich C, Coupland SE, Kapp A, Schmitt-Gräff A, Buchen R, Witschel H (2001). Long term outcome of ocular adnexal lymphoma subtyped according to the REAL classification. Br J Ophthalmol.

[3] Moslehi R, Coles FB, Schymura MJ (2011). Descriptive epidemiology of ophthalmic and ocular adnexal non-Hodgkin's lymphoma. Expert Rev Ophthalmol.

[4] Harris NL (1991). Extranodal lymphoid infiltrates and mucosa-associated lymphoid tissue (MALT). A unifying concept. Am J Surg Pathol.

[5] Stefanovic A, Lossos IS (2009). Extranodal marginal zone lymphoma of the ocular adnexa. Blood.

[6] Mombaerts I, Rose GE, Garrity JA (2016). Orbital inflammation: Biopsy first. Surv Ophthalmol.

[7] Pelloski CE, Wilder RB, Ha CS, Hess MA, Cabanillas FF, Cox JD (2001). Clinical stage IEA - IIEA orbital lymphomas: outcome in the era of modern staging and treatment. Radiother Oncol.

[8] Kwon M, Lee JS, Lee C, Yoon DH, Sa HS (2021). Prognostic factors for relapse and survival among patients with ocular adnexal lymphoma: validation of the eighth edition of the American Joint Committee on Cancer (AJCC) TNM classification. Br J Ophthalmol.

